# Supplementary material for: Plant AtEH/Pan1 proteins drive autophagosome formation at ER-PM contact sites with actin and endocytic machinery
Source: Nat Commun. 2019 Nov 13;10:5132. doi: 10.1038/s41467-019-12782-6 (PMC6853982; doi:10.1038/s41467-019-12782-6)
Supplement: Supplementary file 1 — Supplementary Information [file 41467_2019_12782_MOESM1_ESM.pdf]

Plant AtEH/Pan1 proteins drive autophagosome formation at ER-PM contact sites with actin and endocytic machinery.

Wang and Pleskot et al., Supplementary information.

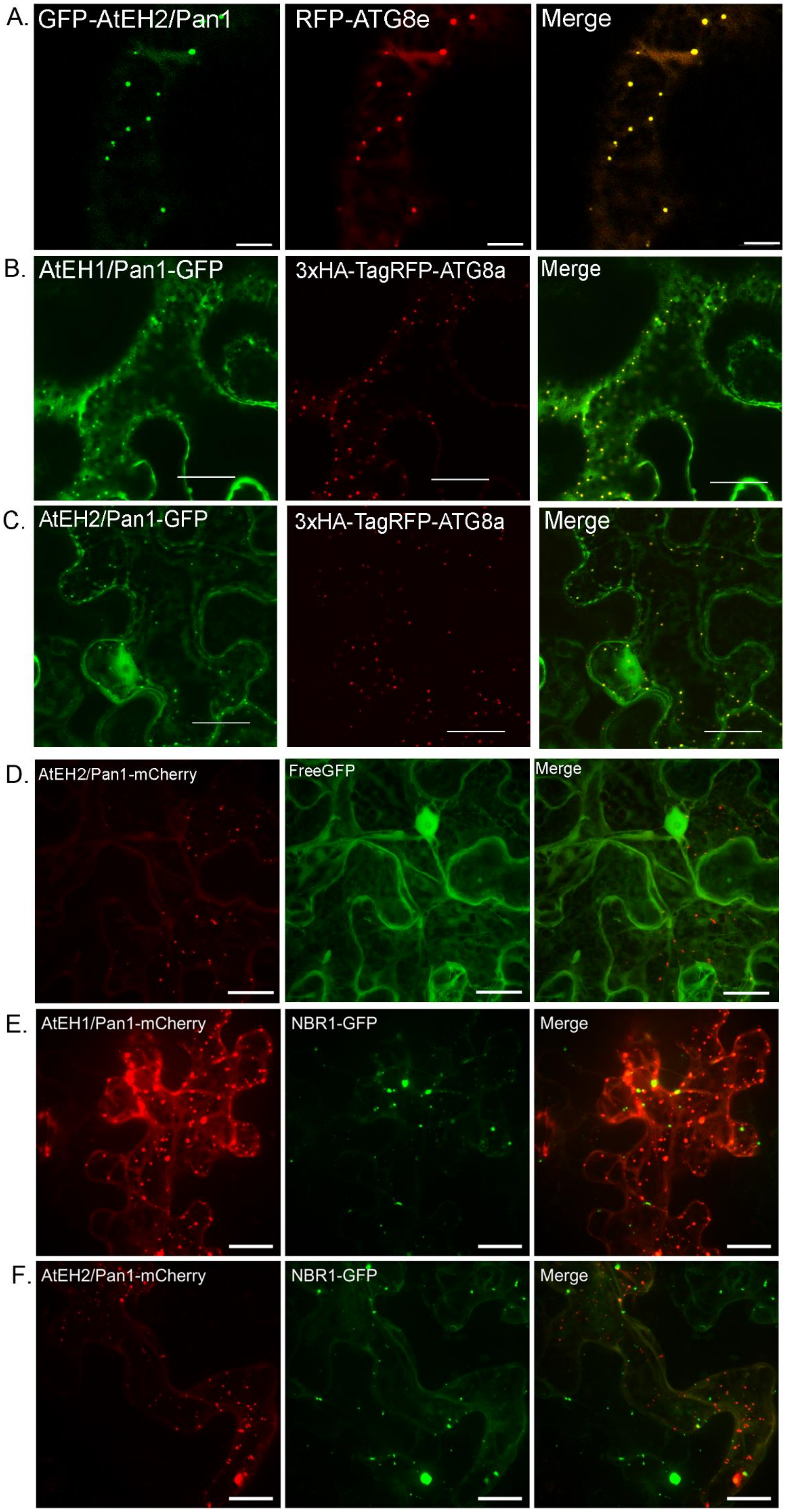
A.
GFP-AtEH2/Pan1
RFP-ATG8e
Merge
B.
AtEH1/Pan1-GFP
3xHA-TagRFP-ATG8a
Merge
C.
AtEH2/Pan1-GFP
3xHA-TagRFP-ATG8a
Merge
D.
AtEH2/Pan1-mCherry
FreeGFP
Merge
E.
AtEH1/Pan1-mCherry
NBR1-GFP
Merge
F.
AtEH2/Pan1-mCherry
NBR1-GFP
Merge

Supplementary Figure 1. AtEH/Pan1 co-localizes with autophagosome markers in epidermal *N. benthamiana* epidermal cells. A. Similar to AtEH1/Pan1, RFP-AtEH2/Pan1 co-localizes with the autophagosome marker, ATG8 in transient expression assays in *N. benthamiana* (Scalebars equal 10 μm). **B-C.** Both AtEH1/Pan1-GFP and AtEH2/Pan1-GFP co-localize with ATG8 at autophagosomes, indicating that both N- or C- terminal GFP fusions do not affect the protein localization. **D.** As a control, AtEH2/Pan1-mCherry was co-expressed with cytosolic GFP, and no recruitment of free GFP into the autophagosomes was identified. **E and F**. NBR1-GFP, a marker that labels ubiquitinated cargo and aggresomes, co-expression with both AtEH/Pan1 proteins resulted in partial co-localization with AtEH1/Pan1-GFP (E), whereas hardly any co-localization with AtEH2/Pan1-GFP could be observed (F), indicating differences between both proteins (Scalebars equal 20 μm).

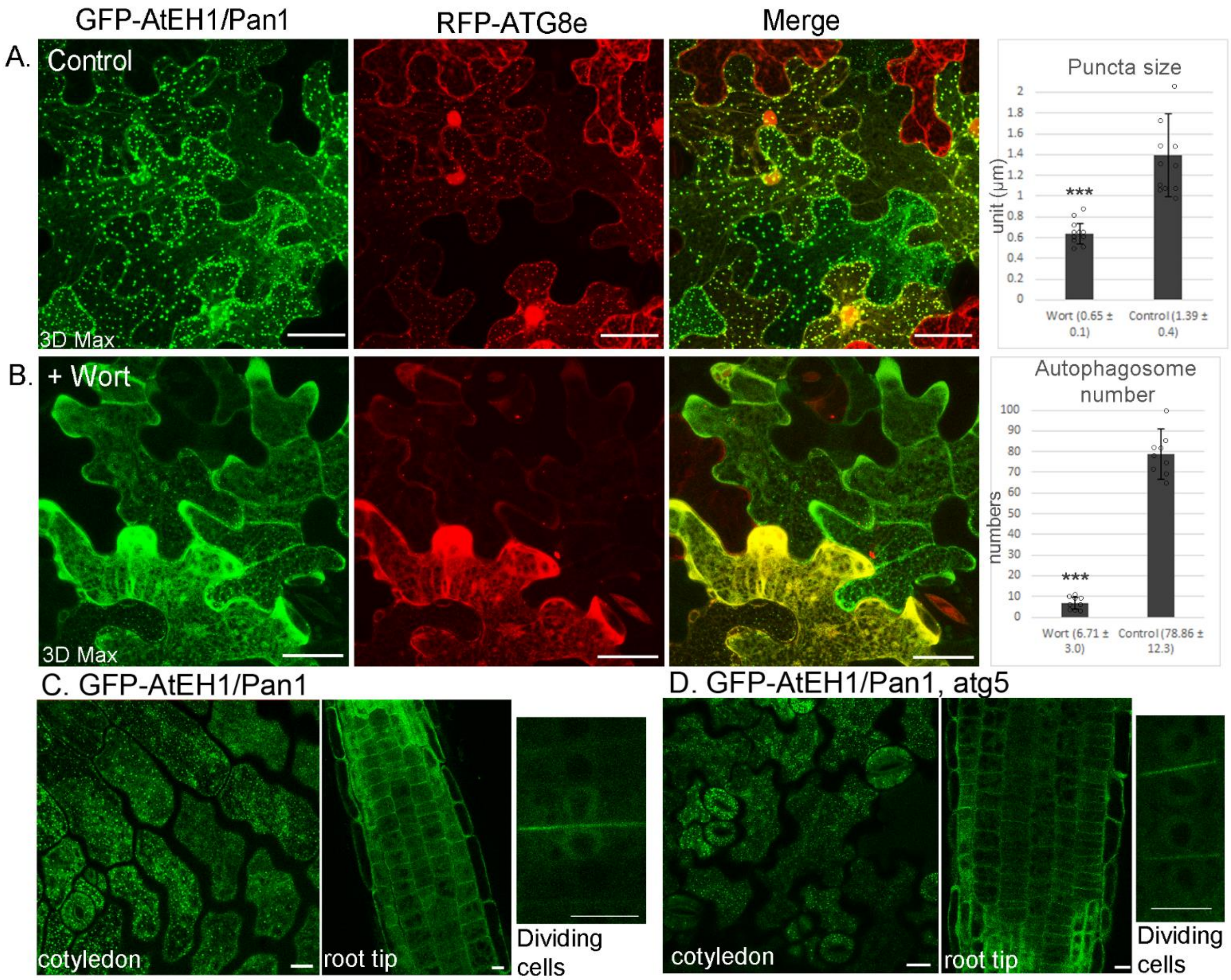

Supplementary Figure 2. Drug treatments of GFP-AtEH1/Pan1 expressing cells, further confirm its localization to autophagosomes. A and B. Wortmannin, a PI3K blocker that inhibits autophagy, treatment reduces the number of autophagosomes (calculated as a number of ATG8e-labelled punctae) and affects the size of the GFP-AtEH1/Pan1 punctae (measured from an area of 50 x 50µm, n=10). **C and D.** The GFP-AtEH1/Pan1 localization in either wild type Arabidopsis (*Col-0*) or in the autophagy deficient mutant *atg5*, is similar, i.e. punctate-labelling in cotyledons and PM-labelling in roots (also see Figure2), indicating that the localization of AtEH1/Pan1 is independent of the autophagy machinery (Scalebars equal 10 µm). Error bars are S.D, ***P < 0.001 in Student's T-test.

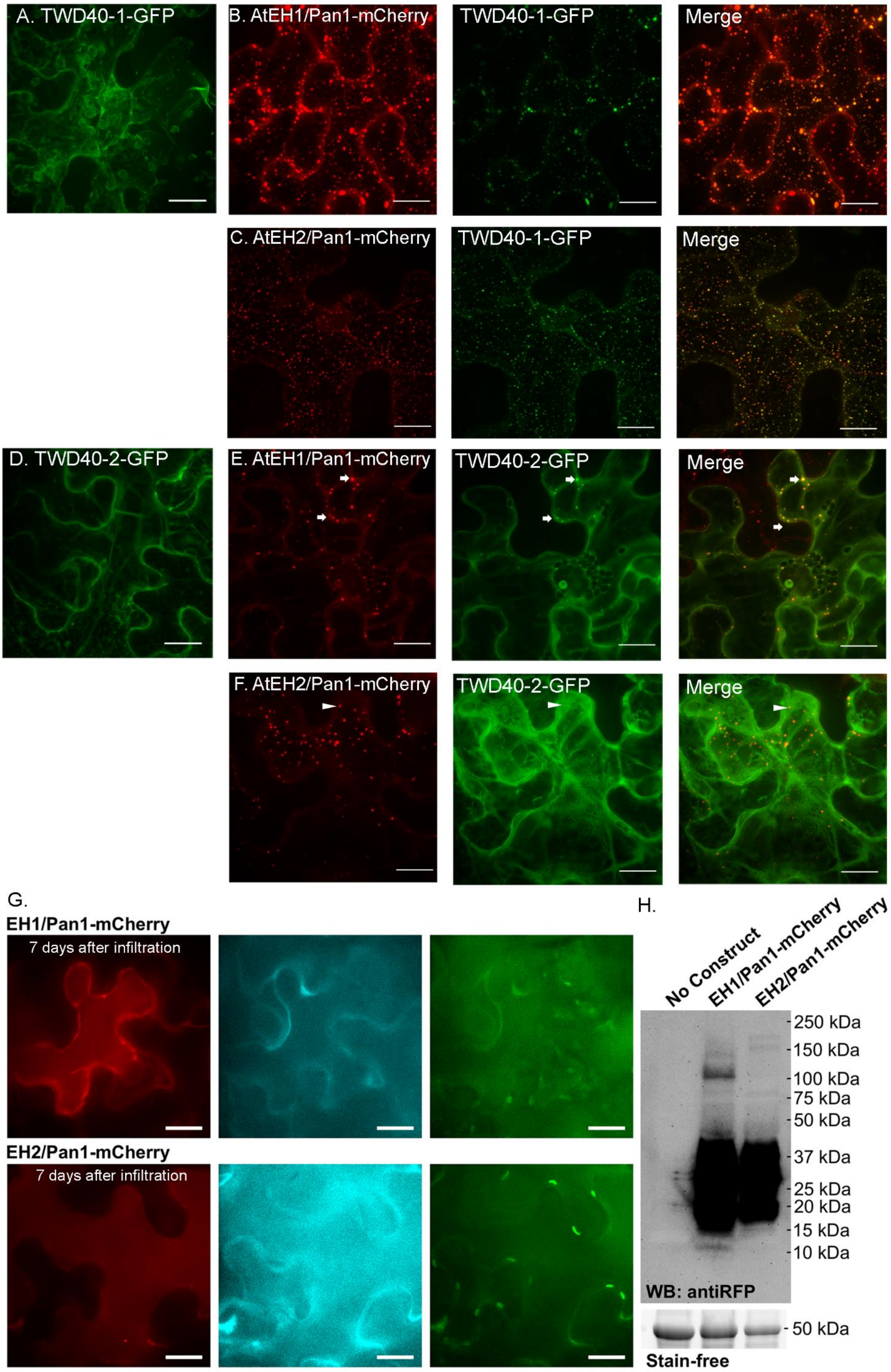

A. TWD40-1-GFP
B. AtEH1/Pan1-mCherry
TWD40-1-GFP
Merge
C. AtEH2/Pan1-mCherry
TWD40-1-GFP
Merge
D. TWD40-2-GFP
E. AtEH1/Pan1-mCherry
TWD40-2-GFP
Merge
F. AtEH2/Pan1-mCherry
TWD40-2-GFP
Merge
G.
EH1/Pan1-mCherry
7 days after infiltration
EH2/Pan1-mCherry
7 days after infiltration
H.
No Construct
EH1/Pan1-mCherry
EH2/Pan1-mCherry
250 kDa
150 kDa
100 kDa
75 kDa
50 kDa
37 kDa
25 kDa
20 kDa
15 kDa
10 kDa
WB: antiRFP
50 kDa
Stain-free

Supplementary Figure 3. AtEH/Pan1 localizes to autophagosomes where it is able to recruit other subunits of the TPLATE complex. A-F. Representative images of epidermal *N. benthamiana* cells transiently expressing AtEH/Pan1 fused to mCherry together with various other TPLATE complex subunits. **A-F.** In contrast to single infiltrations (A and D) where TWD40-1 and TWD40-2 localize to the cytoplasm upon over-expression in *N. benthamiana*, co-expression of AtEH1/Pan1-mCherry recruits the TWD40-1 and TWD40-2 subunits to the autophagosomes (B and E). Interestingly, AtEH2/Pan1-mCherry is only able to recruit TWD40-1 (C) but not TWD40-2 (F) onto the autophagosomes, indicating there is a mechanism of selectivity in the recruitment of different TPLATE subunits, and some subunits might require other subunits for their recruitment to the autophagosomes. Arrows in panels E and F point to either recruitment of TWD40-2 to AtEH1/Pan1 and to the absence of recruitment of TWD40-2 to AtEH2/Pan1. **G.** Strong vacuole labelling was found after pro-longed expression of AtEH/Pan-mCherry, indicating that these proteins are subject to vacuolar degradation. The signal is only detected in the red channel (570-625nm), but not in the blue (454-496nm) or the green channel (500-550nm), indicating that the signal does not originate from autofluorescence of dying cells (Scalebars equal 20 µm). **H.** Western blot analysis of total protein extract from *N. benthamiana* expressing EH/Pan1-mCherry after 7 days shows that protein is still present but heavily degraded.

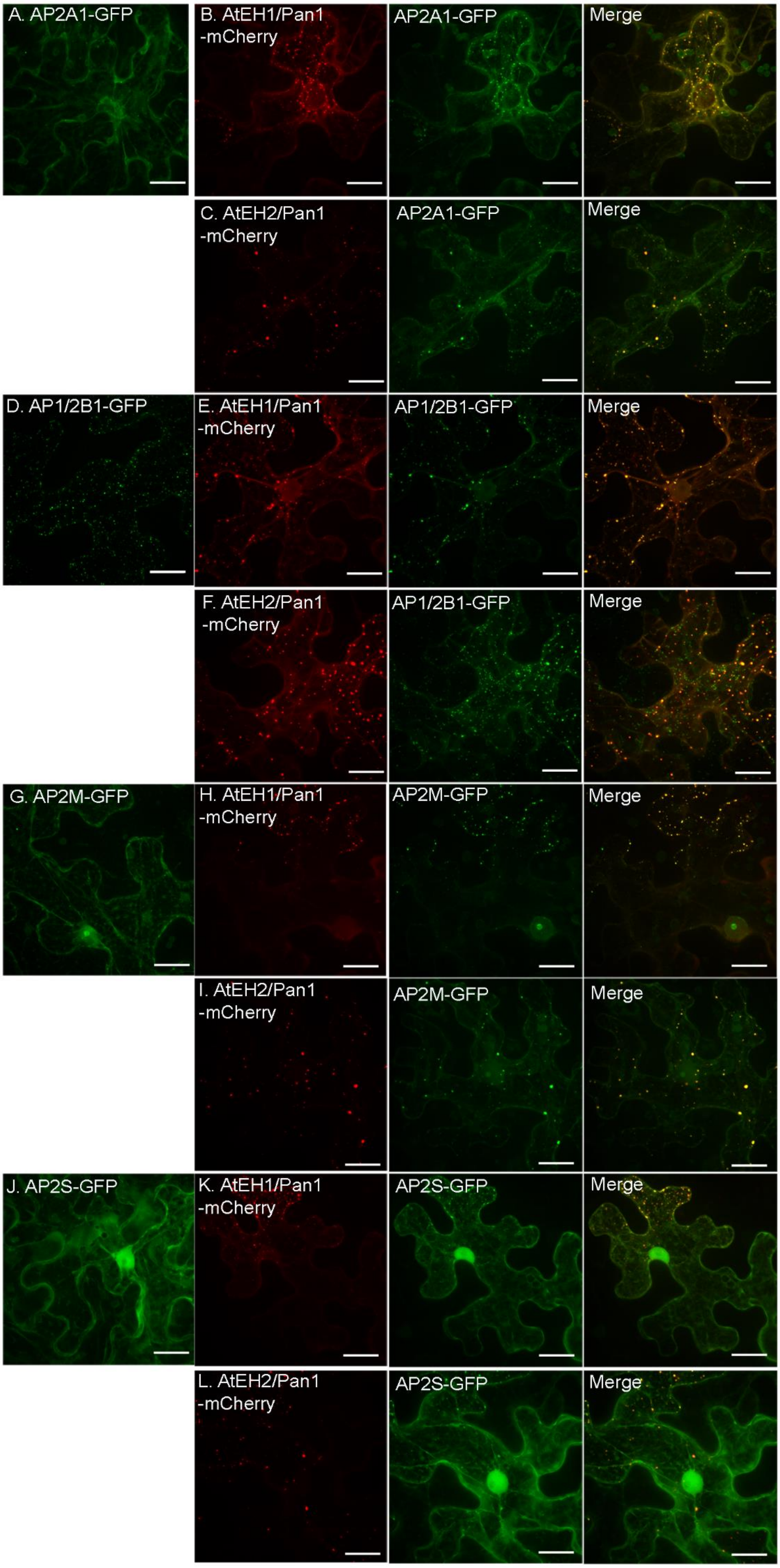
A. AP2A1-GFP
B. AtEH1/Pan1 -mCherry
AP2A1-GFP
Merge
C. AtEH2/Pan1 -mCherry
AP2A1-GFP
Merge
D. AP1/2B1-GFP
E. AtEH1/Pan1 -mCherry
AP1/2B1-GFP
Merge
F. AtEH2/Pan1 -mCherry
AP1/2B1-GFP
Merge
G. AP2M-GFP
H. AtEH1/Pan1 -mCherry
AP2M-GFP
Merge
I. AtEH2/Pan1 -mCherry
AP2M-GFP
Merge
J. AP2S-GFP
K. AtEH1/Pan1 -mCherry
AP2S-GFP
Merge
L. AtEH2/Pan1 -mCherry
AP2S-GFP
Merge

Supplementary Figure 4. AtEH/Pan1 recruit subunits of the AP-2 complex onto the autophagosomes. All of the AP-2 subunits were recruited to the AtEH/Pan1 positive autophagosomes. These includes AP2A1 (**A-C**), AP1/2B1 (**D-F**), AP2M (**G-I**) and AP2S (**J-L**) (Scalebars equals 20 µm).

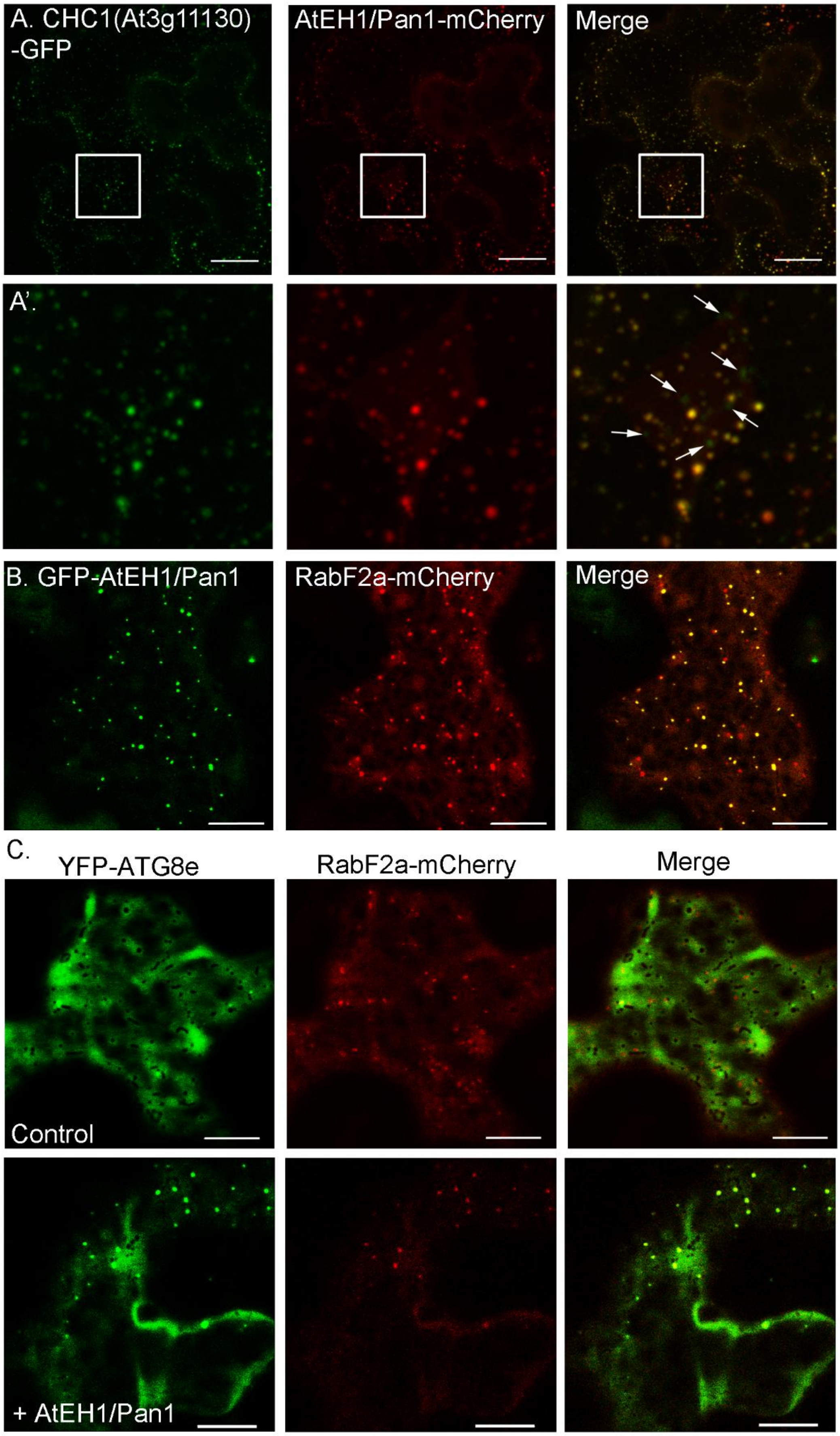

Supplementary Figure 5. Strong co-localization is identified between AtEH/Pan1 and endosome markers. A-A'. Clathrin heavy chain, CHC1, localizes to the TGN/EE (arrows) and also partially co-localizes with AtEH1/Pan1 positive autophagosomes (Scalebar equals 20 μm). **B.** Similarly, strong co-localization is also observed between AtEH1/Pan1 and RabF2a-mCherry, a commonly used late endosomal marker. **C.** In cells transformed with the

autophagosome marker YFP-ATG8e and the late endosomal marker RabF2a-mCherry, hardly any autophagosomes can be observed. However, in the presence of AtEH1/Pan1, strong co-localization between ATG8e and RabF2a is observed (Scalebar equals 10 µm).

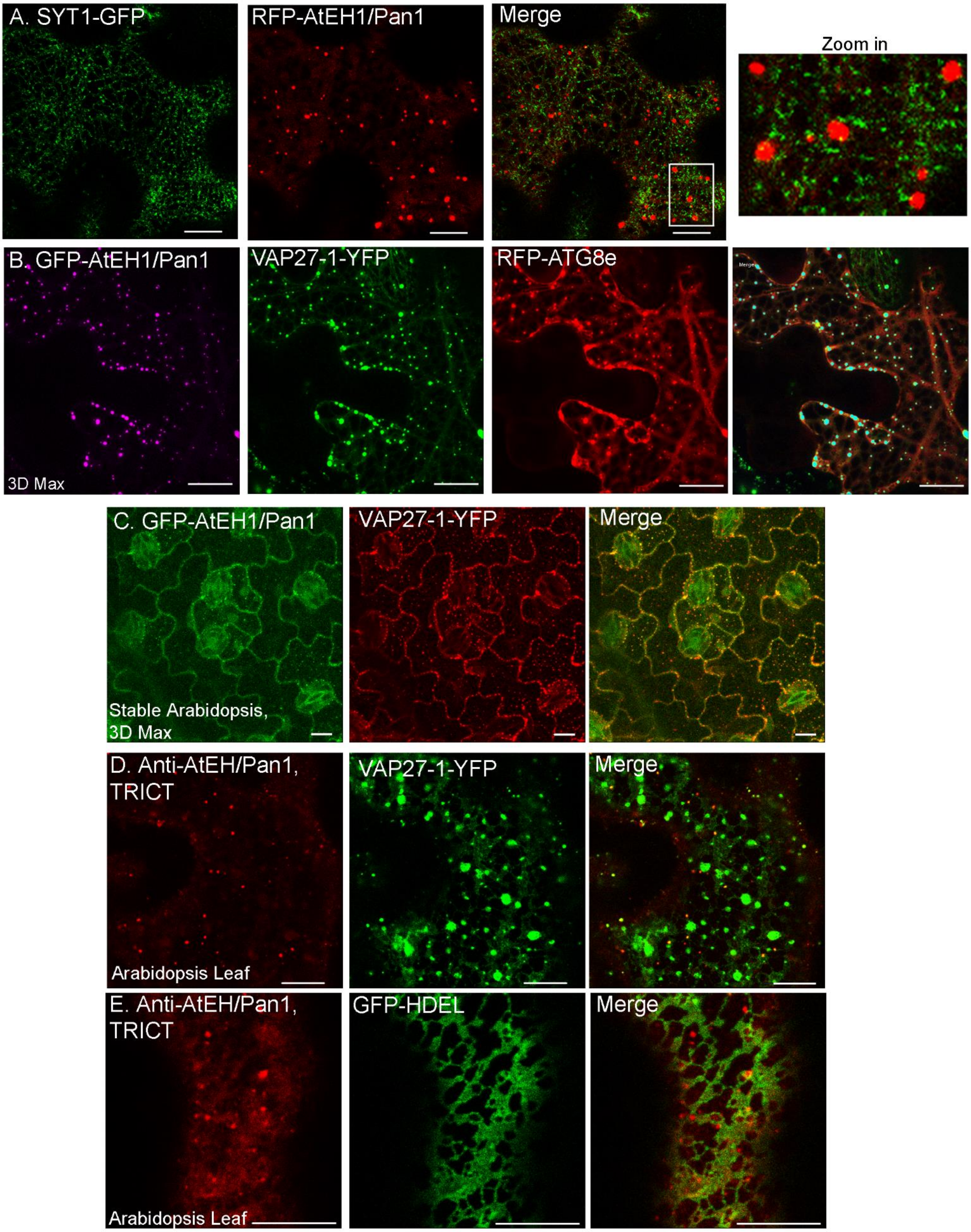

Supplementary Figure 6. AtEH1/Pan1 labelled autophagosomes are found at ER-PM contact sites. A. Strong association but little co-localization is seen between RFP-AtEH1/Pan1 with another EPCS resident protein, SYT1-GFP when both are transiently co-expressed in *N. benthamiana*. **B.** Triple expression of GFP-AtEH1/Pan1, RFP-ATG8e and VAP27-1-YFP in *N. benthamiana* leaf epidermal cells, showing that the three proteins co-localize at the same structures. **C.** The association between AtEH1/Pan1 with EPCS was further confirmed using an Arabidopsis line

expressing VAP27-1-YFP and GFP-AtEH1/Pan1. AtEH1/Pan1 labelled autophagosomes are found to co-localise with VAP27-1 at the ER-PM contact sites. **D.** Similarly, immunofluorescence was performed using anti-AtEH/Pan1 in cells of a VAP27-1-YFP expressing Arabidopsis line. Endogenous AtEH/Pan1 was found to co-localize with VAP27 labelled EPCS. **E.** As a control, immunolocalization of anti-AtEH/Pan1 was performed in Arabidopsis plants expressing GFP-HDEL. Red punctate structures were found to be associated with the ER network, but did not co-localize with discrete punctae in the green channel, indicating that our labelling is specific (Scalebar equals 10 μm).

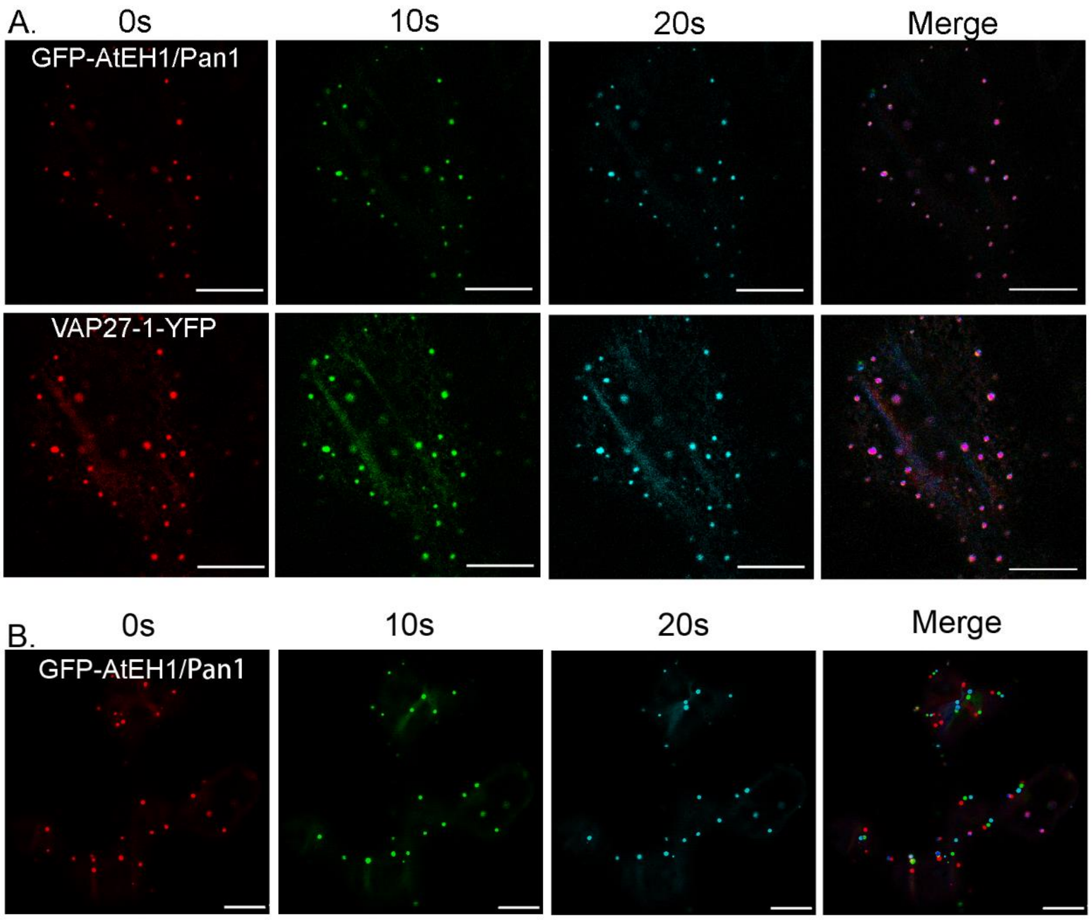

Supplementary Figure 7. The ER-PM contact sites associated AtEH1/Pan1 autophagosomes are stationary. **A.** Time series of a cell co-expressing GFP-AtEH1/Pan1 and VAP27-1-YFP over 20 seconds. Each picture represents a time point and is pseudo-coloured to represent the different time frames; red (0 seconds), green (15 seconds) and cyan (30 seconds). The merged picture indicates that the punctae are persistent (magenta), indicating that EPCS and AtEH1/Pan1 puncta are stationary. **B.** In contrast, the majority of GFP-AtEH1/Pan1 labelled autophagosomes are much more mobile when VAP27-1 is not over-expressed (Scalebar equals 10 µm).

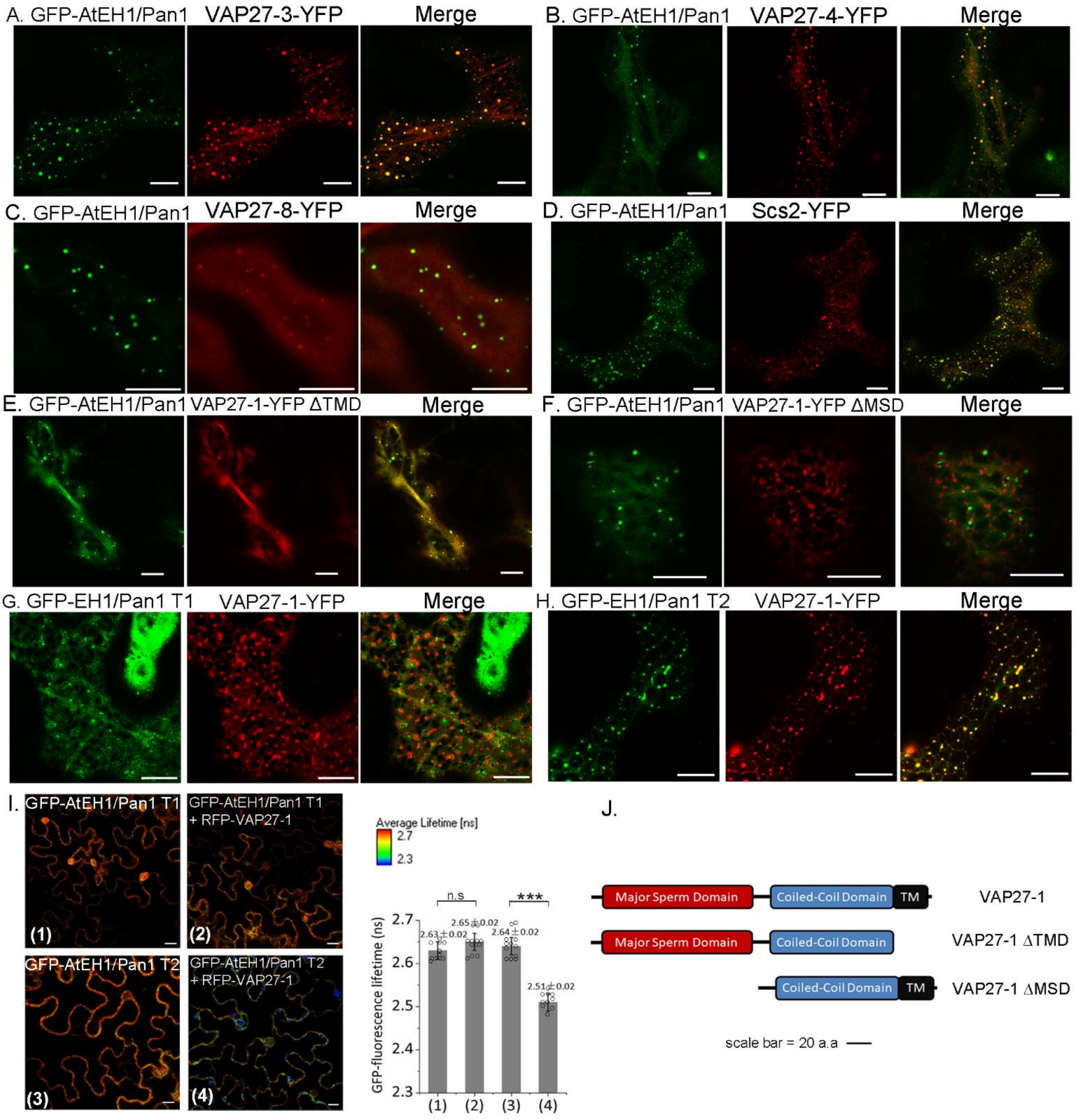

Supplementary Figure 8. GFP-AtEH1/Pan1 co-localizes with multiple VAP27 proteins at punctate structures. A-H. Transient co-expression of AtEH1/Pan1 proteins together with members of the VAP family in *N. benthamiana*. Scalebars equal 10 µm. **A-B.** GFP-AtEH1/Pan1 co-expressed with two ER and EPCS localized VAP27 proteins, VAP27-3-YFP and VAP27-4-YFP. Strong co-localization is found at the EPCS. **C.** VAP27-8-YFP is also recruited to the GFP-AtEH1/Pan1 puncta when the two proteins are co-expressed. **D.** Similar co-localization is also found when a yeast homologue of VAP27, Scs2-YFP is used. **E-F.** GFP-AtEH1/Pan1 is co-expressed with two VAP27-1 truncations, VAP27-1 ΔTMD and VAP27-1 ΔMSD. Only VAP27-1 ΔTMD can to some extent be recruited to AtEH1/Pan1 puncta, indicating that the interaction between the two proteins requires the major sperm domain (MSD). **G-H.** On the other hand, when full length VAP27-1 is co-expressed with either, GFP-AtEH1/Pan1-T1 or GFP-AtEH1/Pan1-T2, only the C-terminus of

AtEH1/Pan1 is able to co-localize with VAP27-1, suggesting the interaction requires the C-terminal sequence in AtEH1/Pan1. I. Using FRET-FLIM, only the lifetime of GFP-AtEH1/Pan1 T2 is significantly reduced when combined with RFP-VAP27-1, indicating interaction between VAP27 and the C-terminal domain of AtEH1/Pan1. The fluorescence life-time of the GFP-AtEH1/Pan1 T1 or T2 (donor only control) is on average 2.63 ± 0.02ns and 2.64 ± 0.02ns, respectively (1) and (3); the life-time of AtEH1/Pan1 T1 combined with RFP-VAP27-1 is 2.65 ± 0.02ns (2), and the life-time of AtEH1/Pan1 T2 combined with RFP-VAP27-1 is 2.51 ± 0.02ns (3). **J. Schematic illustration of the various VAP27-1 mutants used in this study** (Scalebars equal 10 µm). N ⩾ 10 for every FRET-FLIM analysis. Error bars are S.D., ***P < 0.001 in Student's T-test.

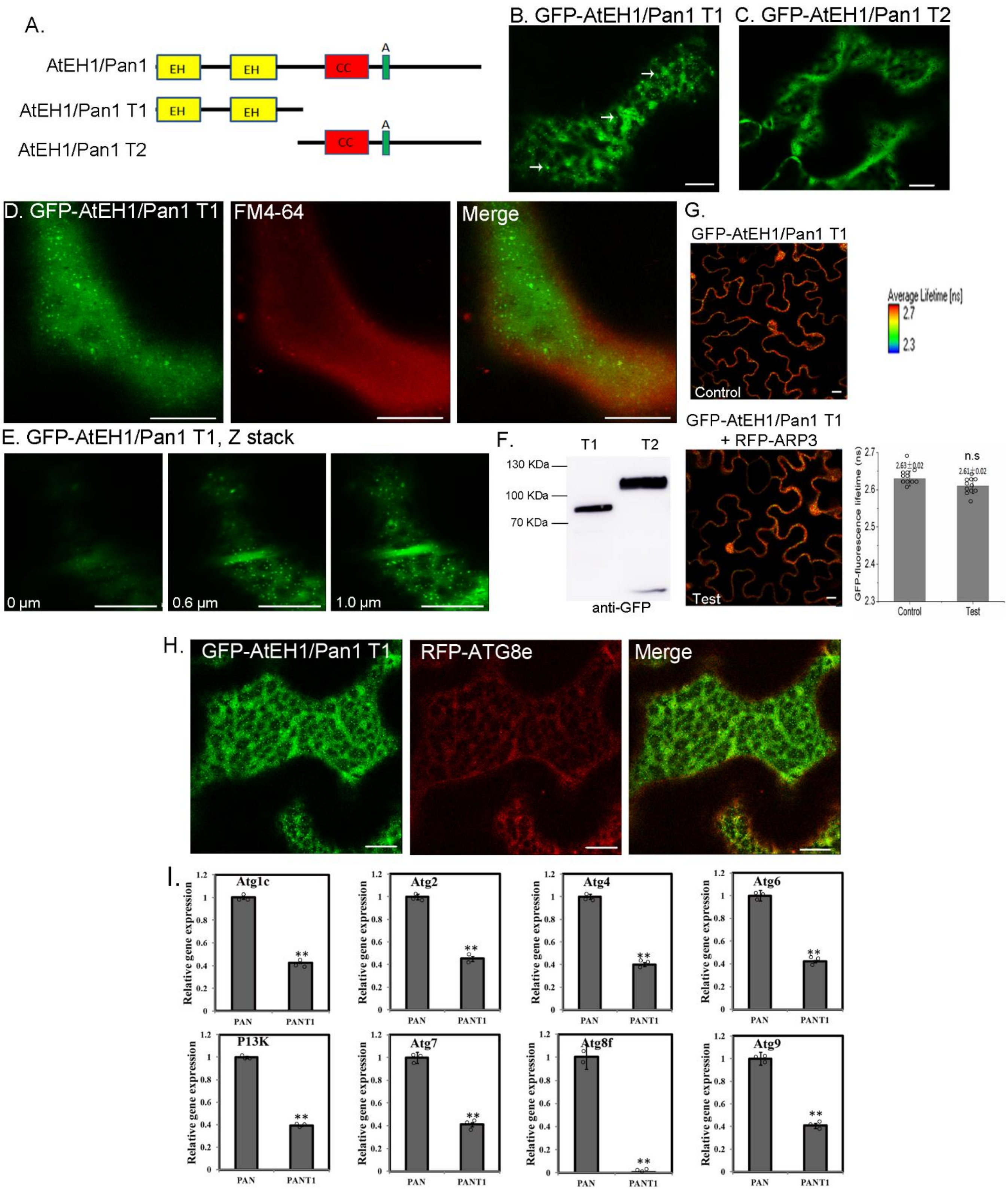

Supplementary Figure 9. Expression of AtEH1/Pan1 domain deletion mutants does not induce autophagy. **A.** Schematic illustration of the different AtEH1/Pan1 truncations used in this study. **B and C.** When transiently expressed in *N. benthamiana*, the GFP-AtEH1/Pan1 T1 truncation (a.a.1-500, containing two EH domains) localizes to the cytoplasm and to punctae, likely endomembrane structures, while the AtEH1/Pan1 T2 truncation (a.a.474-1019, without EH domains) mainly localizes to the cytoplasm. **D.** GFP-AtEH1/Pan1 T1 localized to punctate structures that are found at the same focal plane as the FM4-64 labelled PM. **E.** Z-stack of a cell

expressing GFP-AtEH1/Pan1 T1 showing that the punctae reside close to the PM focal plane. **F.** Western-blot analysis of a *N. benthamiana* leaf extract expressing GFP-AtEH1/Pan1 T1 and T2 truncations showing that both proteins are made and stable **G.** Using FRET-FLIM, the fluorescence life-time of the GFP-AtEH1/Pan1 T1 donor protein is on average 2.63 ± 0.02ns, very similar to its lifetime when co-expressed with RFP-ARP3, indicating there is no interaction between these two proteins (Scalebars equal 10 µm). **H.** Transient expression of GFP-AtEH1/Pan1 T1 truncation, does not induce the formation of autophagosomes in contrast to the full-length protein. The T1 construct mostly localizes to the cytosol as well as to small punctate structures without co-localization with ATG8. **I.** Moreover, autophagic activity in AtEH1/Pan1 T1 expressing cells, as measured by the relative expression of various ATG genes, is lower compared to the situation when the full-length protein is expressed. N ⩾ 11 for every FRET-FLIM analysis. Error bars are S.D., n.s. = not significant, $P > 0.05$, $^{**}P < 0.01$ in Student's T-test.

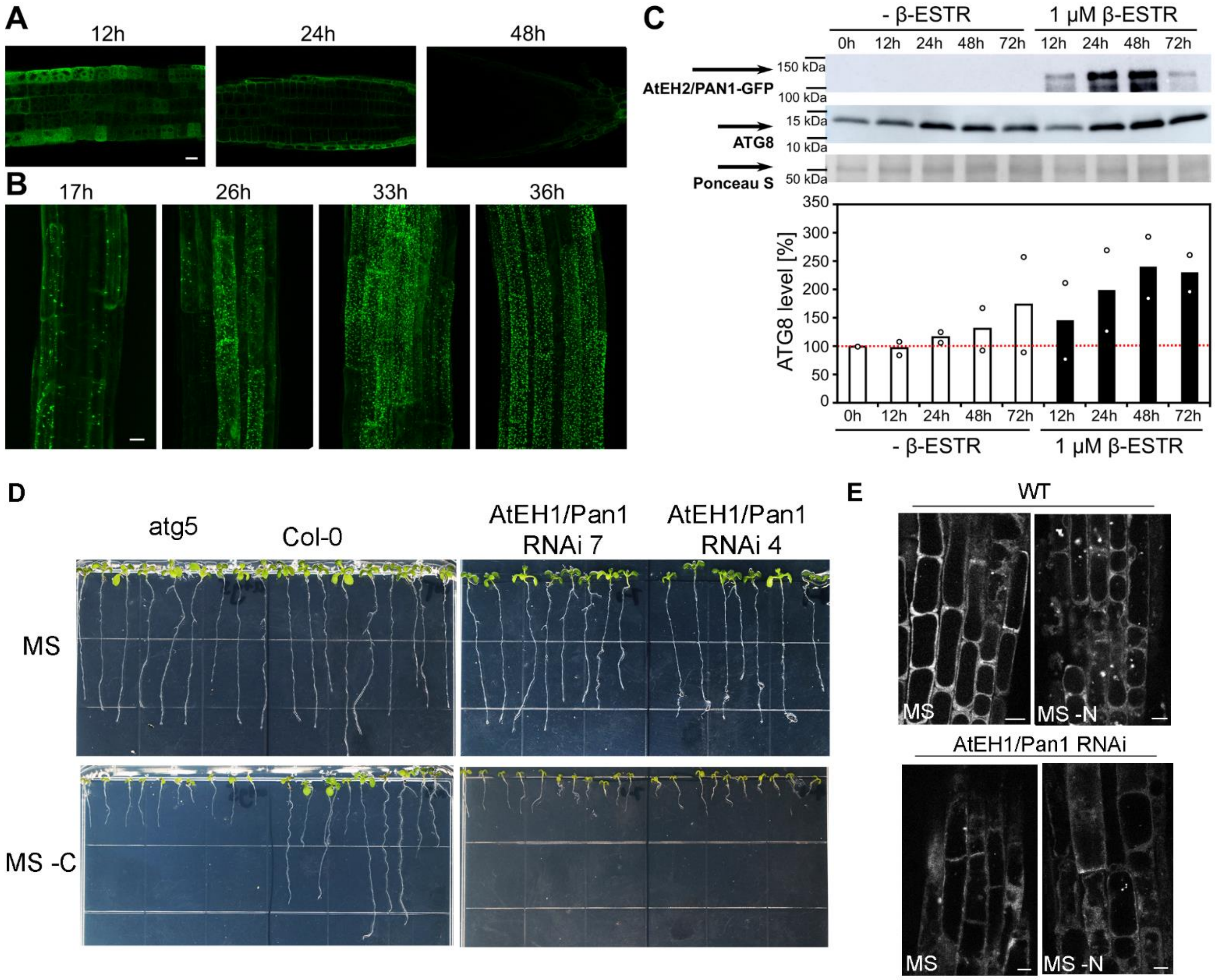

Supplementary Figure 10. Induced over expression of AtEH2/Pan1 boosts autophagy. A and B. Confocal images of root meristems (B) and maximum projections of root differentiation/maturation zone cells (C) of seedlings taken at the respective time points after transfer to Estradiol-containing medium. 12hrs post induction, clear cytoplasmic signal can be detected and the first autophagosomes appear in the differentiation/maturation zone. At 24hrs post induction, the signal in the meristem cells is predominantly membrane-associated and autophagosomes in the differentiation/maturation zone are clearly present up to 36hrs post induction. At 48hrs post induction, the signal in the meristem zone is strongly decreased. **C.** After induction by transfer, AtEH2/Pan1 protein is detected after 12 hours and increases up to 48hrs. ATG8 protein levels also increase up to 48hrs. ATG8 levels of transferred seedlings to non-induced and induced conditions are plotted using Ponceau S staining as normalization factor. The plot shows the average of two technical repeats of a typical result from at least two biological replicas (Scale bars equal 15 µm). **D.** Two independent AtEH1/Pan1 RNAi lines show retarded root growth in nutrient depleted medium (MS -C) compared to the wild type plants. The autophagy deficient mutant, *atg5*, was used as a control to demonstrate the effectiveness of the starvation treatment. **E.** At normal growth conditions, autophagosomes (labelled by MDC) are rarely detectable in either WT or AtEH1/Pan1 RNAi mutant lines, however their number in WT plants was increased during nitrogen starvation, but not in AtEH1/Pan1 mutant (Scale bars equal 10 µm).

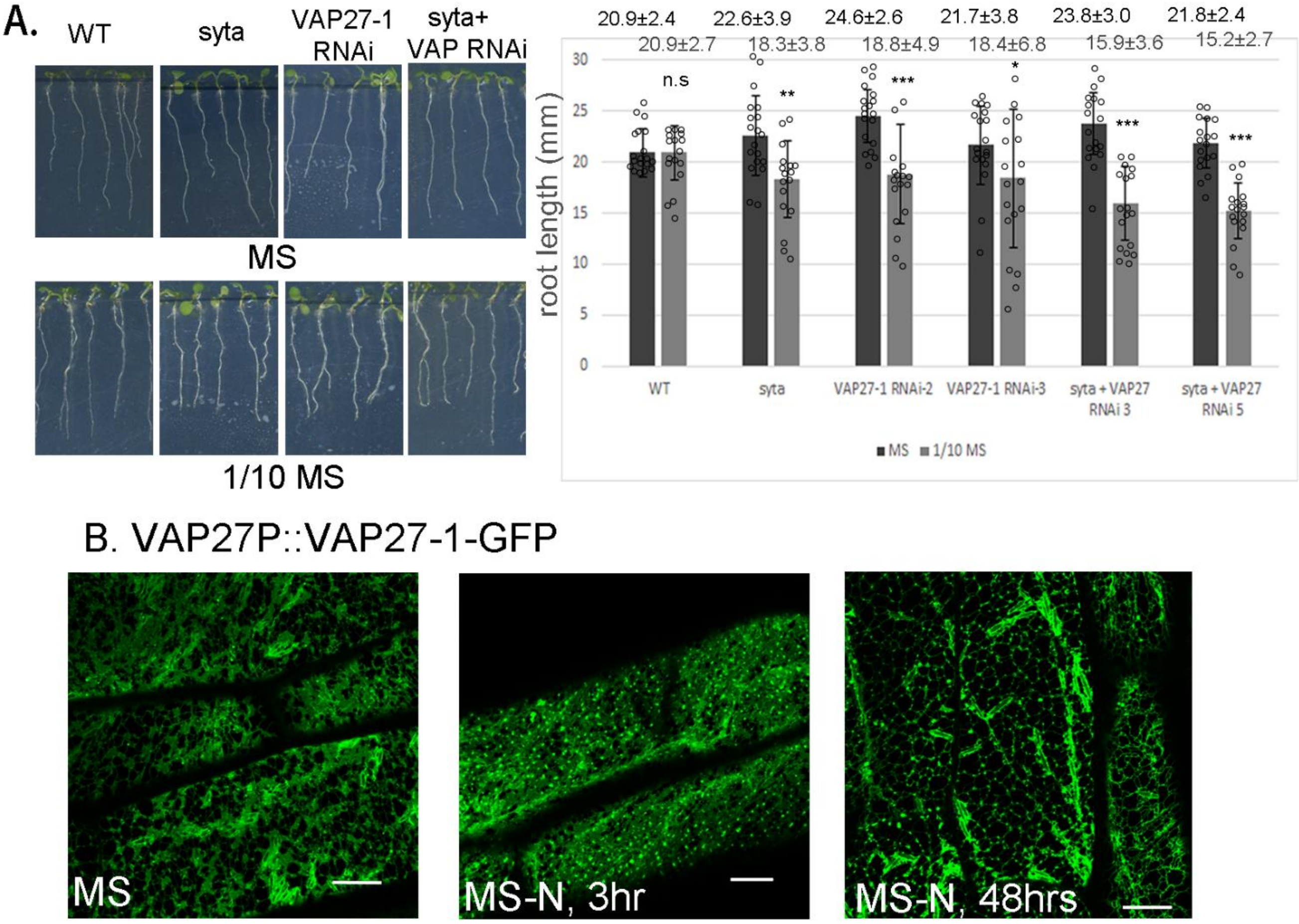

Supplementary Figure 11. Plant ER-PM contact sites are involved in starvation induced autophagy. A. Plants with knock-out or knock-down expression of EPCS genes are also defective in autophagy. Root growth in the *syta* mutant, VAP27 RNAi lines, and *syta* + VAP27 RNAi double mutant was analysed. Under low nutrient conditions (1/10MS), mutant lines showed reduced growth compared to their development in high nutrient conditions (MS), whereas little effect on growth was observed for wild type plants (n =19). **B.** In Arabidopsis hypocotyl cells, VAP27-1-GFP is mainly localized to the ER network, with some EPCS being labelled. Enhanced ER-PM contact site labelling of VAP27-1 is found when cells are starved for 3 hours, suggesting an enhanced ER-PM interaction is required when cells are under autophagic stress (Scalebars equal 10 μm). Error bars are S.D., (* P < 0.05, ** P <0.01, *** P <0.001 in Student's T-test).

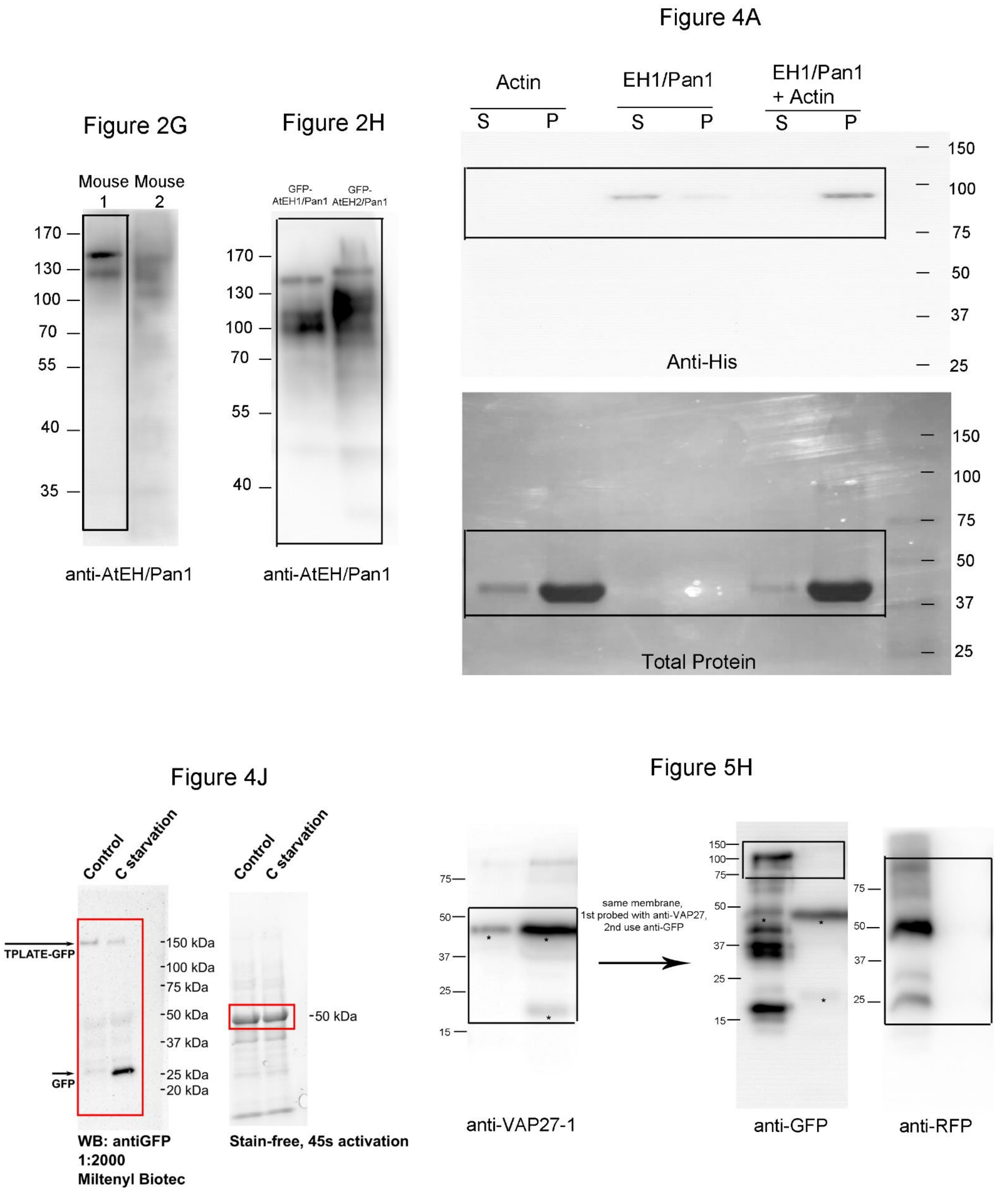

Supplementary Figure 12. Uncropped blot images shown in Figure 2, Figure 4 and Figure 5. Please note in figure 5H, the blot was first probed with anti-VAP27-1, and the same blot was used with different antibody. Protein bands labelled in asterisk are detected in the first experiment, and were also visible in the second experiment.

Figure S3H

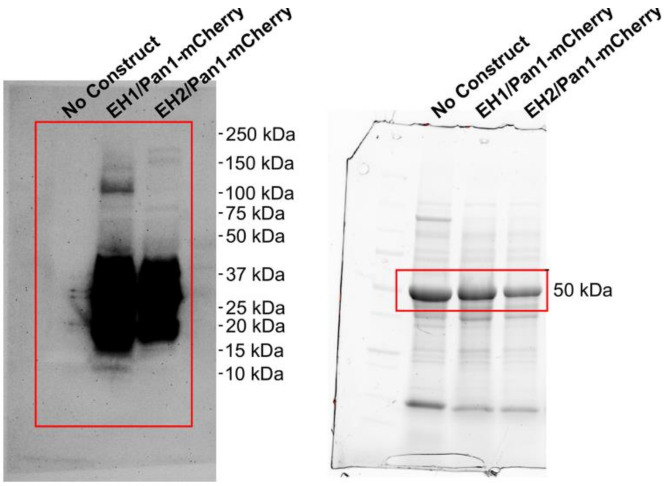

WB: 6G6 anti-RFP, 1:2000, Chromotec

Stain-free, 45s activation

Figure 6D

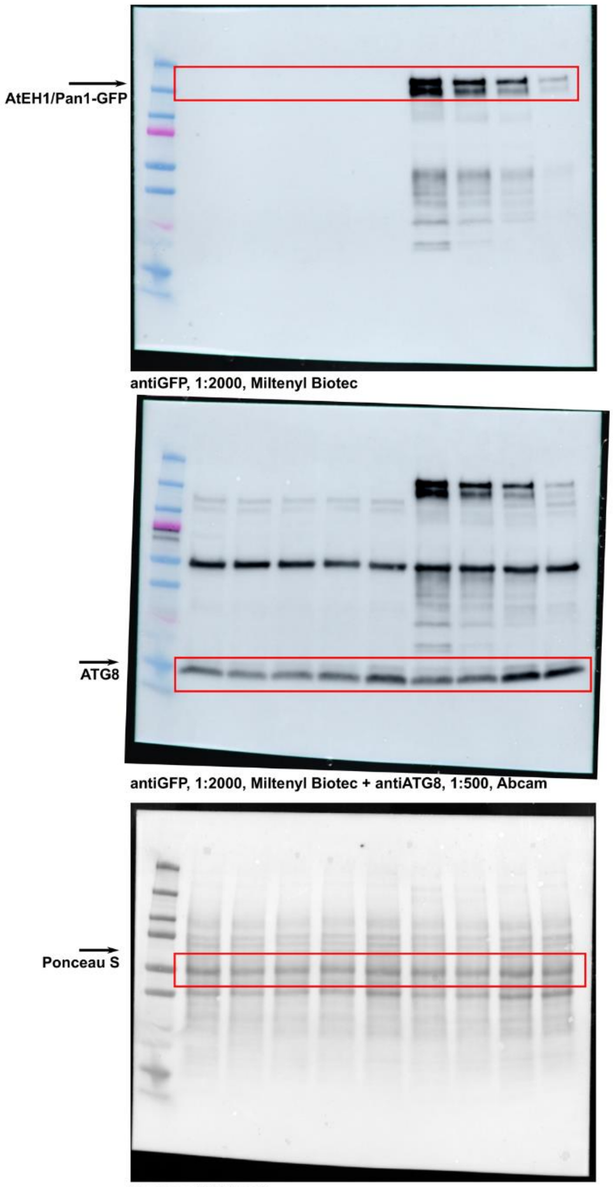

Ponceau S (Sigma)

Figure S10C

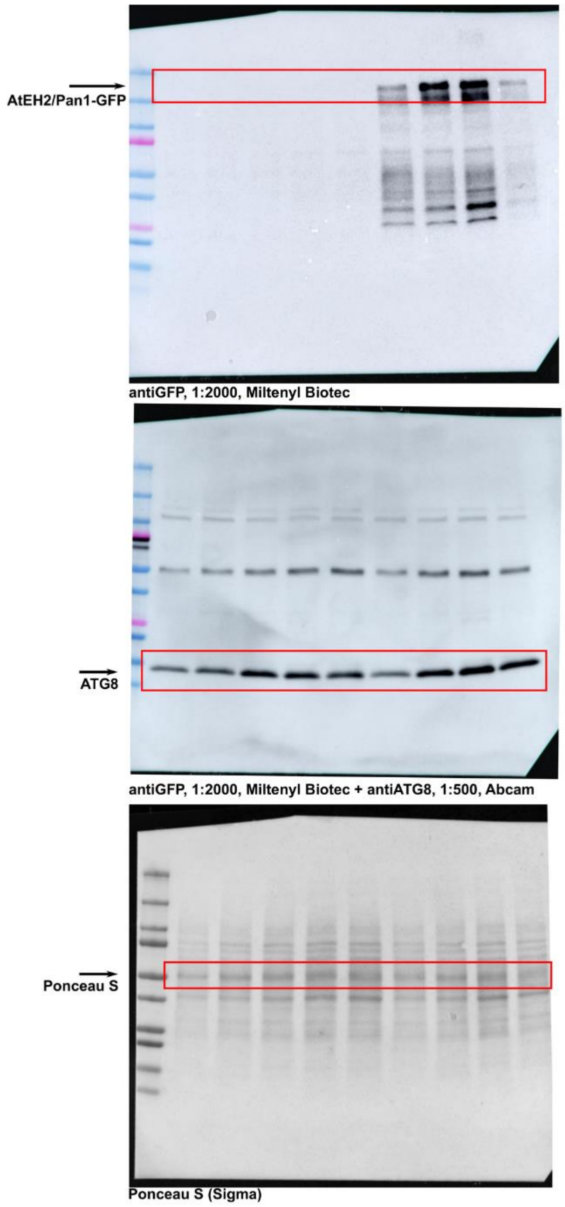

Ponceau S (Sigma)

Supplementary Figure 13. Uncropped blot images shown in Figure 6, Figure S3 and Figure S10.

Supplemental Table 1. The list of primers used for cloning and genotyping

Gene	F primer	R primer
AtEH1/Pan1	ggggacaagtttgtacaaaaaagcaggcttcccgcca ATGGCGGGTCAGAATCCTAAC	ggggaccactttgtacaagaaagctgggtcCTAGGTCAG ATAACTGGA ACTCCTTC
AtEH2/Pan1	ggggacaagtttgtacaaaaaagcaggcttcccgccaAT GGCAGCACCAAGGCCGA	ggggaccactttgtacaagaaagctgggtcCTAGAAAGC ATTCCAATTATCAGAAC
AtEH1/Pan1 T1	ggggACAAgTTTgTACAAAAAAgCAgg CTTCACCATGGCGGGTCAGAATCCT AACATGG	ggggACCACTTTgTACAAgAAAgCTgggTCC TAACCACTGCCAGGTTGAGGGC
AtEH1/Pan1 T2	GAATTCGCGGCCGCATGGGTGCGC GGCCAATCA	GAG TGC GGC CGC CTA GAA GGA GTT CCA GTT ATC
AtEH1/Pan1 GST	CAA CAG GAGCTC GCG GGT CAG AAT CCT AAC AT	CAA CAG CTCGAG CTA GAA GGA GTT CCA GTT ATC TGA C
AtEH1/Pan1 antigen	CAACAGGCTAGCAGAAGCCCCTTTA TGTTCGA	CAACAGAAGCTTCTAGAAAGCATTCCAA TTATCAGAAC
mRuby3	GGGGACAAGTTTGTACAAAAAAGC AGGCTCAatggtgtctaagggcgaagag	GGGGACCACTTTGTACAAGAAAGCTGG GTActtgtacagctcgtccatgc
SALK_083997. 1	LP: TGCAGCAGAAATTGTTCTGG	RP: ATGAGACCACCAGTTCCTGC
LBaI 1.3	ATTTTGCCGATTTCGGAAC	

Supplemental Table 2. The list of primers used for real-time qPCR

Species	Gene	F primer	R primer
Arabidopsis Real-time	*ACTIN1*	CATCAGGAAGGACTTGTACGG	GATGGACCTGACTCGTCATAC
	Pan1p	GTCGCTTTCTGATCGGTCAC	TAGCAGCAGGATTAGGTCGG
	ATG7	TCTATGACCCGTGTCACCTT	GAGGCTTGACCAACAAGAGA
	ATG8a	CAAGCTTGGAGCTGAGAAAG	GCAACGGTAAGAGATCCAAA
	ATG8b	GAATCAAGCTTGGAGCTGAA	TGTAGAGAAACCCGTCTTCG
	ATG8c	CTTTCAAGTTGGAACACCCA	CATACACAAATTGCCCAACA
	ATG8d	TATGTTGTACGGAAGCGGAT	AGAAACCCGTCTTCGTCTTT
	ATG8e	GTACCTTGTGCCATCAGACC	AGGAAGCCATCTTCGTCTTT
	ATG8f	AGTACCTAGTCCCGGCTGAT	AGGAAGAACATTGTCCACGA
	ATG8h	ATCTGCCAGACATGGAGAAG	CAAAGAGAGCTTTGGATGGA
	ATG18a	TGTTTCTCAGGGTGTTGGTT	TGAGAGCGAAGCAAGCTATT
N. benthamiana Real-time	*eIF4a*	CCCAGAGAGGAAATACAGTG	CAATAGACGGACCAGATTCG
	NbATG1c	TGGAAAGTCCCTCATCTGCACCTG	GCCTGCCTACCTCAACCTTCTCAT
	NbATG2	GCAATTGGGCTTGGAGTGCATTTG	CCTGTCGGGCATCTCTAGGTTGAT
	NbATG3	GGAAACTGACAATCTTATAG	GACATCCTCAAGTACAAGC
	NbATG4	GGCGAAGCTGACTGGATACCTGTT	ATCATCCTGCACGCCGACAATGTA
	NbATG6	ACCTGCGTAAAGGAGTTTGCTGAC	AGAGCTTTGGTCCAACTTTCCTGC
	NbATG7	CCAGCAGTGGAAGCAGAAGGTCTT	GCCACCGACTTTCCCGTGTATCA
	NbPI3K	GCTGTGCTGGTTACTCCGTCATC	ACTGACTTTCCGCTCCACCCATA
	NbATG8f	GACAAGAAAAAGTATCTCGTG	GTGTTTTCTCCACTGTAAGTAA
	NbATG9	TCCGGTGGATTTGCTGCTGTTCT	ACATCGCTCCCTGTGGGTCAAG

Supplemental Table 3. List of published fluorescent protein (FP) markers used in this study

Name	Details	Reference
RFP-ATG8e	FP marker for autophagosomes	Wang et al., 2016a
YFP-ATG8e	FP marker for autophagosomes	Wang et al., 2016a
RFP-HDEL	FP marker for endoplasmic reticulum	Wang et al., 2014
YFP-Actin-Cb	FP marker for actin cytoskeleton	Wang and Hussey, 2017
RFP-ARP3	A protein from ARP2/3 complex	Wang et al., 2016a
VAP27-1-YFP	An ER-PM contact site resident protein	Wang et al., 2014
Scs2-YFP	Yeast homologue of VAP27-1	Wang et al., 2014
VAP27-3-YFP	A protein from the VAP27 family; ER, EPCS localization	Wang et al., 2016b
VAP27-4-YFP	A protein from VAP27 family; ER, EPCS localization	Wang et al., 2016b
VAP27-8-YFP	A protein from VAP27 family; PM localization	Wang et al., 2016b
SYT1-GFP	An ER-PM contact site resident protein	Siao et al., 2016
RabF2a-mCherry	FP marker for late endosomes	Geldner et al., 2009
35S::AtEH1-Pan1-GFP	FP marker for the AtEH1/Pan1 (At1g20760) TPC subunit	Gadeyne et al., 2014
35S::AtEH2/Pan1-GFP	FP marker for the AtEH2/Pan1 (At1g21630) TPC subunit	Gadeyne et al., 2014
35S::TPLATE-GFP	FP marker for the TPLATE (At3g01780) TPC subunit	Van Damme et al., 2004
UBQ10::YFP-ATG8a	FP marker for autophagosomes	Ortiz-Morea, F. A. et al. (2016).
UBQ10::mCherry-ATG8e	FP marker for autophagosomes	Gift from Professor Yasin Dagdas
35S::NBR1-GFP	FP marker for ubiquitinated proteins and aggresomes	Dagdas et al., Elife 2018
UBQ::3xHA-TagRFP-AtAtg8a	FP marker for autophagosomes	Gift from Professor S.P. Dinesh-Kumar
