# Supplementary material for: Plant AtEH/Pan1 proteins drive autophagosome formation at ER-PM contact sites with actin and endocytic machinery
Source: Nat Commun. 2019 Nov 13;10:5132. doi: 10.1038/s41467-019-12782-6 (PMC6853982; doi:10.1038/s41467-019-12782-6)
Supplement: Supplementary file 3 — Description of Additional Supplementary Files [file 41467_2019_12782_MOESM3_ESM.pdf]

Description of Additional Supplementary Files

File Name: Supplementary Movie 1
Description: List of sequences used to generate the phylogenetic tree of Figure 1.

File Name: Supplementary Data 1
Description: Homotypic fusion of GFP-AtEH1/Pan1 labelled autophagosomes in leaf epidermal cells.
