# Supplementary material for: Plant AtEH/Pan1 proteins drive autophagosome formation at ER-PM contact sites with actin and endocytic machinery
Source: Nat Commun. 2019 Nov 13;10:5132. doi: 10.1038/s41467-019-12782-6 (PMC6853982; doi:10.1038/s41467-019-12782-6)
Supplement: Supplementary file 6 — Reporting Summary [file 41467_2019_12782_MOESM6_ESM.pdf]

nature research

Corresponding author(s): NCOMMS-18-35683-A; Patrick Hussey

Reporting Summary

Nature Research wishes to improve the reproducibility of the work that we publish. This form provides structure for consistency and transparency in reporting. For further information on Nature Research policies, see Authors & Referees and the Editorial Policy Checklist.

Statistical parameters

When statistical analyses are reported, confirm that the following items are present in the relevant location (e.g. figure legend, table legend, main text, or Methods section).

n/a	Confirmed	
☐	☒	The exact sample size (*n*) for each experimental group/condition, given as a discrete number and unit of measurement
☐	☒	An indication of whether measurements were taken from distinct samples or whether the same sample was measured repeatedly
☐	☒	The statistical test(s) used AND whether they are one- or two-sided<br>*Only common tests should be described solely by name; describe more complex techniques in the Methods section.*
☒	☐	A description of all covariates tested
☒	☐	A description of any assumptions or corrections, such as tests of normality and adjustment for multiple comparisons
☐	☒	A full description of the statistics including central tendency (e.g. means) or other basic estimates (e.g. regression coefficient) AND variation (e.g. standard deviation) or associated estimates of uncertainty (e.g. confidence intervals)
☒	☐	For null hypothesis testing, the test statistic (e.g. *F*, *t*, *r*) with confidence intervals, effect sizes, degrees of freedom and *P* value noted<br>*Give P values as exact values whenever suitable.*
☒	☐	For Bayesian analysis, information on the choice of priors and Markov chain Monte Carlo settings
☒	☐	For hierarchical and complex designs, identification of the appropriate level for tests and full reporting of outcomes
☒	☐	Estimates of effect sizes (e.g. Cohen's *d*, Pearson's *r*), indicating how they were calculated
☐	☒	Clearly defined error bars<br>*State explicitly what error bars represent (e.g. SD, SE, CI)*

Our web collection on statistics for biologists may be useful.

Software and code

Policy information about availability of computer code

Data collection: All software used in this study are either commercially available or open source.

Data analysis: All software used in this study are either commercially available or open source.

For manuscripts utilizing custom algorithms or software that are central to the research but not yet described in published literature, software must be made available to editors/reviewers upon request. We strongly encourage code deposition in a community repository (e.g. GitHub). See the Nature Research guidelines for submitting code & software for further information.

Data

Policy information about availability of data

All manuscripts must include a data availability statement. This statement should provide the following information, where applicable:

- Accession codes, unique identifiers, or web links for publicly available datasets
- A list of figures that have associated raw data
- A description of any restrictions on data availability

The relevant data supporting the findings of this study are available from the corresponding author.

Field-specific reporting

Please select the best fit for your research. If you are not sure, read the appropriate sections before making your selection.

☒ Life sciences ☐ Behavioural & social sciences ☐ Ecological, evolutionary & environmental sciences

For a reference copy of the document with all sections, see nature.com/authors/policies/ReportingSummary-flat.pdf

Life sciences study design

All studies must disclose on these points even when the disclosure is negative.

Sample size	Sufficient sample sizes were chosen for each experiment to determine whether the outcome was statistically significant. At least two independent transgenic lines were used for every relevant study, and at least three repetitions were performed for every studies.
Data exclusions	No data were excluded from this study.
Replication	We confirmed that all studies performed here is reproducible in all replications.
Randomization	Experimental samples were selected randomly during the experiment without any pre-judgment.
Blinding	Blinding was not implemented in this study.

Reporting for specific materials, systems and methods

Materials & experimental systems

n/a	Involved in the study	
☐	☒	Unique biological materials
☐	☒	Antibodies
☒	☐	Eukaryotic cell lines
☒	☐	Palaeontology
☐	☒	Animals and other organisms
☒	☐	Human research participants

Methods

n/a	Involved in the study	
☒	☐	ChIP-seq
☒	☐	Flow cytometry
☒	☐	MRI-based neuroimaging

Unique biological materials

Policy information about availability of materials

Obtaining unique materials	all unique biological materials are readily available from the authors

Antibodies

Antibodies used	anti-ATG8 antibodies (Agrisera and Abcam); anti-HisTag (Abcam); anti-GFP HRP conjugated (Miltenyi Biotec); anti-Tubulin (Abcam); TRITC or FITC-conjugated secondary antibodies (Jackson ImmunoResearch); HRP-conjugated rabbit/rat/mouse secondary antibody (GE Healthcare); Alexa Fluor® FluoroNanogold™ Fab' goat-anti-mouse IgG (Nanoprobes, USA); anti-RFP (Chromotec 6G6-20).
Validation	All commercial antibodies were validated by the suppliers. ATG4, NAP1, VAP27-1 antibodies were validated in previous publications, and AtEH/Pan1 antibody is validated in this study.

Animals and other organisms

Policy information about studies involving animals; ARRIVE guidelines recommended for reporting animal research

Laboratory animals	n/a
Wild animals	n/a

Field-collected samples | n/a
